# Health Maxims

**Published:** 1879-01

**Authors:** 


### HALL'S JOURNAL OF HEALTH.

**Our Legitimate Scope is almost boundless: for whatever begets pleasurable and harmless feelings, promotes Health; and whatever induces disagreeable sensations, engenders Disease.**

WE AIM TO SHOW HOW DISEASE MAY BE AVOIDED, AND THAT IT IS BEST, WHEN SICKNESS COMES, TO TAKE NO MEDICINE WITHOUT CONSULTING A PHYSICIAN.

**Vol. 26 ] JANUARY, 1879. [No. 1.**

### HEALTH MAXIMS.

The object of the following maxims is to communicate some generally accepted principles in their application to the preservation of health, and the cure of disease without medicine, in short phrase, few words and disconnected sentences; to be taken up and laid down at a moment's notice, on steamship, tramway, packet or rail car, at such odds and ends of time as fall to the lot of travelers and others, and which else might not be appropriated so usefully, because in this age of restlessness and hurry the care of the health, like the search for religion, is considered one of the things which can be dispensed with, until a more convenient season in the future. It is hoped that some who would not spend the time to hear a lecture or read a book may be enticed to peruse a paragraph now and then in reference to the care of the body which, in being put into practice, may have an important bearing in the prolongation of life.

